# Medication Use among Pregnant Women from the 2015 Pelotas (Brazil) Birth Cohort Study

**DOI:** 10.3390/ijerph17030989

**Published:** 2020-02-05

**Authors:** Bárbara Heather Lutz, Vanessa Iribarrem Avena Miranda, Marysabel Pinto Telis Silveira, Tatiane da Silva Dal Pizzol, Sotero Serrate Mengue, Mariângela Freitas da Silveira, Marlos Rodrigues Domingues, Andréa Dâmaso Bertoldi

**Affiliations:** 1 Faculty of Medicine, Department of Social Medicine & Post-Graduate Program in Epidemiology, Federal University of Pelotas, Rua Marechal Deodoro, 1160, Pelotas CEP 96020-220, RS, Brazil; 2 Post-Graduate Program in Epidemiology, Federal University of Pelotas, Rua Marechal Deodoro, 1160, Pelotas CEP 96020-220, RS, Brazil; vanessairi@gmail.com (V.I.A.M.); mariangelafreitassilveira@gmail.com (M.F.d.S.); andreadamaso.epi@gmail.com (A.D.B.); 3 Institute of Biology, Department of Physiology and Pharmacology & Post-Graduate Program in Epidemiology, Federal University of Pelotas, Rua Marechal Deodoro, 1160, Pelotas CEP 96020-220, RS, Brazil; marysabelfarmacologia@gmail.com; 4 Post-Graduate Program in Epidemiology, Federal University of Porto Alegre, Av. Ipiranga, 2752, Sala 203, Porto Alegre CEP 90610-000, RS, Brazil; tatiane.silva@ufrgs.br (T.d.S.D.P.); sotero@ufrgs.br (S.S.M.); 5 Post Graduate Program in Physical Education & Post-Graduate Program in Epidemiology, Federal University of Pelotas, Rua Marechal Deodoro, 1160, Pelotas CEP 96020-220, RS, Brazil; marlosufpel@gmail.com

**Keywords:** drug use, drug utilization, pharmaceutical preparations, self-medication, pharmacoepidemiology, pregnancy, cohort studies

## Abstract

*Background:* Medication use during pregnancy is a common practice that has been increasing in recent years. The aim of this study is to describe medication use among pregnant women from the 2015 Pelotas (Brazil) Birth Cohort Study. *Methods:* This paper relies on a population-based cohort study including 4270 women. Participants completed a questionnaire about the antenatal period, including information about medication use. We performed descriptive analyses of the sample and the medications used and adjusted analyses for the use of medications and self-medication. *Results:* The prevalence of medication use was 92.5% (95% CI 91.7–93.3), excluding iron salts, folic acid, vitamins, and other minerals. The prevalence of self-medication was 27.7% (95% CI 26.3–29.1). In the adjusted analysis, women who had three or more health problems during pregnancy demonstrated higher use of medicines. Self-medication was higher in lower income groups and among smokers and multiparous women (three pregnancies or more). Acetaminophen, scopolamine, and dimenhydrinate were the medications most commonly used. *Conclusions:* This study describes the pattern of drug use among pregnant women in a population-based cohort study, with a high prevalence of self-medication. Greater awareness of the risks of self-medication during pregnancy is required, focusing on groups more prone to this practice, as well as ensuring qualified multidisciplinary prenatal care.

## 1. Introduction

It is estimated that over 90% of pregnant women use prescription and nonprescription medications [1]. The lack of clear information on this subject from both health care providers and mothers results in situations where treatment is discontinued or used at low doses by pregnant women, while, on the other hand, drugs with potentially toxic effects are maintained or started, which makes the issue a public health problem that must be addressed [2]. Therefore, it is necessary to observe the patterns of drug use during the antenatal period to recognize potential improper practices and to guarantee the rational use of medications in pregnancy [3].

The lesson learned from the thalidomide tragedy in the 1960s has led to the recognition that drug use during pregnancy can be very harmful to the fetus [3,4]. It was previously believed that the placenta was a barrier protecting the fetus; today, however, it is known that most drugs cross the placenta [5]. Prescription and over-the-counter drugs, herbal products, dietary supplements, topical creams, inhalers, vitamins, alcohol, nicotine, and illicit drugs can cross the placental barrier and reach the fetal bloodstream [2]. Furthermore, pregnant women very rarely are included in clinical trials of new drugs due to ethical aspects, further justifying the care needed at this stage of life [3,5].

Since 1979, the United States Food and Drug Administration (FDA) drug use risk classification system has been one of the most widely used in the world, which was based on five letter-named risk categories. After their update in 2015, these categories were no longer used, and a reformulation of the content and format of drug package inserts was recommended in United States. This reformulation included a descriptive summary about the risks of medication use during pregnancy and lactation, discussion of data supporting this information and relevant data to assist health care providers in decision-making and counseling of pregnant and lactating women [4].

It is known that the prevalence of chronic diseases, such as hypertension and diabetes, is rising during pregnancy [2] due to the increase in the average age at which women first become pregnant and other factors such as the rise in obesity among the population [4,6]. A study conducted in the United States and Canada showed that over the past three decades, the use of prescription drugs in the first trimester of pregnancy has increased by over 60%, and the use of four or more medications has more than tripled [7]. Current public health policies may also be one of the factors involved in this rising prescription and drug use, such as recommendations for iron and folic acid supplementation [8].

Pelotas is a Southern Brazilian city with a current population of 344,000 inhabitants. Women who gave birth in the city’s hospitals in 1982, 1993, 2004, and 2015 were the target population for four birth cohort studies, constituting what is probably the largest set of birth cohorts in the same geographical location in low- or middle-income countries [9]. The 2015 cohort differed from that of previous studies by recruiting pregnant women during antenatal care.

The present study aims to describe the use of medications among pregnant women from the 2015 Pelotas (Brazil) Birth Cohort according to pharmacological groups and demographic, socioeconomic, and health-related variables, as well as self-medication among this group.

## 2. Materials and Methods

The data from this study are part of the 2015 Pelotas (Brazil) Birth Cohort Study (C2015), conducted in the city of Pelotas, RS, in southern Brazil. All women who gave birth in one of the five maternity wards in Pelotas from 1 January 2015 to 31 December 2015 and who lived in the urban area of the municipality were invited to participate in the study. Methodological details can be found in another publication [9].

This birth cohort study also had an antenatal component. The 123 health facilities and private clinics providing prenatal care in the city were visited weekly, between May 2014 and December 2015, to identify pregnant women with probable delivery dates in 2015. These women were visited at home or invited to go to the research clinic between 16 and 24 weeks of gestation to answer a health questionnaire including questions about medication use. Three types of questionnaires were developed and administered according to the gestational age that the woman presented at the time of the interview.

In the perinatal study, mothers were interviewed at the maternity wards, answering a standardized questionnaire including information about medication use. Since 75% of the participants were also followed up during pregnancy, it was possible to supplement the information on medication use with data captured in the antenatal study.

For drug analysis, a “long” database was constructed (each medication occupying a record line), where the denominator was the total number of drugs reported by pregnant women and collected in C2015 antenatal and perinatal follow-ups. There was no duplication of information in the resulting database as each medication was computed only once within each trimester, even though it was reported in different interviews (antenatal or perinatal follow-up). In the case of disagreement, the information reported at the closest moment to its use was chosen. This strategy made it possible to qualify the information, considering that during pregnancy the recall period is shorter and interviews were conducted at home, which allowed access to prescriptions and the packaging of the medicines used.

Information on medication use was taken from the following question: “Did you use any medication during pregnancy?” If so, the names of the drugs were requested, and subsequently, for each reported medication, the following questions were asked to characterize their use: “Who told you to use it? (the doctor who followed up the pregnancy/another doctor or dentist/another person or yourself)”; “Did you use this medication in the first trimester, that is until the 13th week of pregnancy?”; “For how many days, throughout the first trimester, did you use this medication? (up to 7 days/8 to 14 days/15 to 30 days/31 to 60 days/more than 60 days)”. Similar questions about usage and number of days were also asked regarding the second (14th to 27th week) and third (28th week onwards) gestational trimesters. Women were advised not to consider the use of vitamins and iron salts at the time of the interview. Self-medication was defined when a participant reported having consumed at least one medication by herself or was encouraged to do so by someone other than a doctor or dentist (relative, friend, neighbor, spouse, or partner). 

The drugs were classified into pharmacological groups by the Anatomical Therapeutic Chemical (ATC) classification system [10] at levels 1 (anatomical group), 2 (therapeutic group), and 5 (chemical substance). In this paper, we do not consider the use of iron salts, folic acid, vitamins, and other minerals, because such supplements are routinely recommended to pregnant women and have been analyzed separately in another study [11]. The independent variables were the following: age (≤19, 20–29, 30–47 years), skin color (white, black, and mixed/other), education (0–4, 5–8, 9–11, and 12 or more years of schooling), current income in minimum wages considering the amount of R$ 788.00 (USD 200.00) as one wage in 2015 (≤1, from 1.1 to 3, from 3.1 to 6, from 6.1 to 10, and >10), parity (1, 2, 3, and 4 or more children), number of prenatal visits (less than 6 visits and 6 or more), marital status (with/without a partner), smoking during pregnancy (yes/no), number of chronic and/or acute health problems (none, 1, 2, 3, and 4 or more), hospitalization during pregnancy (yes/no), and trimester of onset of prenatal care (first, second, or third). Age was collected in complete years and subsequently categorized. Skin color (classification commonly used in Brazil to refer to ethnicity [12,13]) was self-reported by women. Schooling was collected based on complete years of study and subsequently categorized. Family income was reported in Brazilian *reais* and later categorized into minimum wages. For parity, we considered the total number of pregnancies, including stillbirths and the current pregnancy.

The variable “trimester of onset of prenatal care” was constructed based on the information contained in the prenatal charts of the study participants, considering the gestational age at the first consultation. This information was available for only 3773 women.

In constructing the variable “number of chronic and/or acute health problems”, the following issues were considered: systemic arterial hypertension, eclampsia or preeclampsia, diabetes mellitus, depression, anemia, threatened abortion, threatened preterm labor, vaginal discharge, vaginal bleeding in the last trimester of pregnancy, urinary tract infection, asthma or bronchitis, thyroid disease, heart disease, tuberculosis, and syphilis or other sexually transmitted diseases such as herpes, gonorrhea, trichomoniasis, genital warts, chlamydia, and condyloma. We chose to categorize the variable into the following categories: no disease, 1, 2, 3, and 4 or more.

Data analysis was performed using the STATA^®^ statistical software, version 12.1 (StataCorp., College Station, TX, USA). The sample was described by presenting the proportions of the independent variables and calculating the frequency of medication use and self-medication according to the independent variables with their respective confidence intervals (95% CI).

We performed adjusted analyses through two regression models, one for assessing medication use and another for self-medication. The analyses were conducted by Poisson regression with robust variance in two hierarchical levels to obtain estimates of prevalence ratios (PR). We maintained in the first level the demographic and socioeconomic variables (skin color, age, education, income, and marital status). In the second level, the variables related to health conditions were included: parity, number of prenatal visits, smoking, number of chronic and/or acute health problems, hospitalization during pregnancy, and trimester of onset of prenatal care. Only variables presenting p-values below 0.20 were kept in the model, ensuring the control for the variables of the same level and the higher level. The level of significance was set at 0.05.

For the analysis of the prevalence of medication use, the total number of women was used as the denominator. For the self-medication analysis, the denominator considered was the number of women who used medications. The differences in the frequency of drug use across trimesters were analyzed using Pearson’s chi-squared test.

The study was approved by the Ethics Committee of the School of Physical Education of the Federal University of Pelotas under protocol 522.064. All interviews were conducted after the participants signed an informed consent form.

## 3. Results

This study used data from 4270 women interviewed in the perinatal study, including those who had stillbirths. Of these, 3949 (92.5%; 95 %CI 91.7–93.3) reported having used some medication during pregnancy. Among these women, 1089 reported having used some drug by self-medication (27.7%; 95% CI 26.3–29.1). Table 1 shows the characteristics of the sample and the prevalence of medication use and self-medication according to the variables studied. Most women interviewed reported having white skin color (70.5%), ages between 20 and 29 years (47.3%), 9 to 11 years of schooling (34.3%), having a family income between 1.1 and 3 minimum wages (47.2%), and having given birth to their first child (49.5%). In addition, most had six or more prenatal visits (85.5%), had a partner (85.5%), did not smoke (83.2%), had four or more health problems during pregnancy (31.2%), had not been hospitalized during this period (80.3%), and had started prenatal care during the first trimester of pregnancy (54.8%).

Medication use was more frequent among white women with 12 years or more of formal education and among those living with a partner. It was also more frequent among pregnant women who had attended six or more prenatal visits, among non-smokers, among those who had been hospitalized, and among those who had begun prenatal care during the first trimester of pregnancy. Medication use was lower among younger women (19 years and under) and among women whose income was up to one minimum wage (Table 1).

The prevalence of self-medication was higher among women who did not have a partner, among those who had less than six prenatal visits, among smokers, and among multiparous women (three pregnancies or more). On the other hand, self-medication was less frequent in more educated women (12 years or more of schooling) with higher income (equal or higher 6.1 minimum wages) and in those who had started prenatal visits in the first trimester of pregnancy (Table 1).

**Figure 1:**
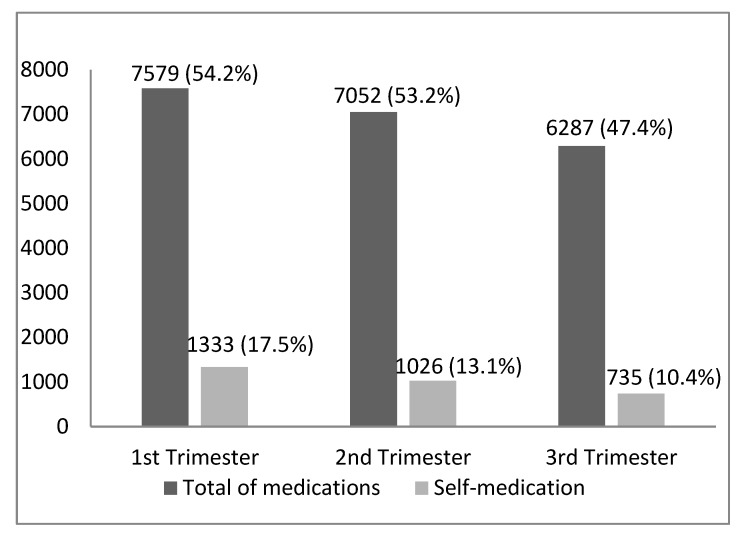
Total of medications used and consumed by self-medication * in each gestational trimester. Pelotas (Brazil) Birth Cohort 2015 (*N* = 14,064 medications). * Excluding iron salts, folic acid, vitamins, and other minerals. The same drug may have been reported by the interviewee in more than one trimester of pregnancy.

There were 14,064 reported uses of medications by pregnant women. Figure 1 shows medication use by trimester of pregnancy. In the first trimester, 7579 medicines were used (representing 54.2% of the medicines used); in the second trimester, 7052 medicines were used (53.2% of the total); and in the third trimester, 6287 medicines were used (47.4% of the total). The frequencies of the drugs used showed a statistically significant difference between trimesters (*p* < 0.001). The same drug may have been reported by the interviewee in more than one trimester of pregnancy. Self-medication was higher in the first trimester of pregnancy. In this period, 1333 drugs were consumed without a prescription from a doctor or dentist (17.5% of the total used in that trimester). This proportion fell in the following trimesters, corresponding to 13.1% of the drugs used in the second trimester (*N* = 1026) and 10.4% of those used in the third trimester of pregnancy (*N* = 735), a difference that was also statistically significant (*p* < 0.001).

The frequency of duration of medication use did not differ much between gestational trimesters. Most medications were used for up to 7 days (an average of 56.4% of the medications used each trimester). Medicines used from 7 to 14 days corresponded to an average of 13.1% in each trimester, drugs used from 15 to 30 days corresponded to an average of 10.1%, and drugs used from 31 to 60 days corresponded to an average of 6.5%. The frequency of medications used for 60 days or more increased slightly from 13% in the first trimester to 16% in the third trimester (data not shown). The most commonly used drugs for 60 days or more were levothyroxine (*N* = 119 women in the first trimester and *N* = 151 in the third trimester) and methyldopa (*N* = 107 women in the first trimester and *N* = 172 in the third trimester).

Table 2 shows the prevalence ratios, with their respective confidence intervals and p-values, only for the variables that remained associated after adjusted analysis. After adjustment, it was observed that medication use was 14% higher among women with three or more health problems. For self-medication, the following variables were statistically significant: income in minimum wages (about 18% lower self-medication in the higher income group), smoking (27% more self-medication among smokers), and parity (25% more self-medication in the group with three pregnancies). 

Table 3 presents the medications consumed by pregnant women, classified according to the first ATC level and the self-medication frequencies for the most commonly used drugs. The most used classes of drugs, according to ATC level 1, were drugs for the alimentary tract and metabolism (*N* = 3964; 30.9% of the drugs used), drugs for the nervous system (*N* = 3676; 28.6%), and anti-infectives for systemic use (*N* = 2565; 20%). Acetaminophen was the most used medication by pregnant women (*N* = 2654), followed by scopolamine (*N* = 1037) and dimenhydrinate (*N* = 922).

The total number of drugs used by self-medication was 1733 (12.3% of the medicines used). Among these, we want to highlight the groups of drugs used for the musculoskeletal system, as 35.4% were used without medical advice. Antiparasitic products, insecticides, and repellents were the second most used group without medical advice (27.8% of the products used in this category were consumed by self-medication).

The most consumed drug by self-medication was acetaminophen (*N* = 559). The use of the nasal vasoconstrictor naphazoline was also noteworthy. About 53% of the pregnant women who used it did so without medical prescription. About 50% (*N* = 100) of women taking dipyrone (metamizole) also did so without medical advice.

**Figure 2:**
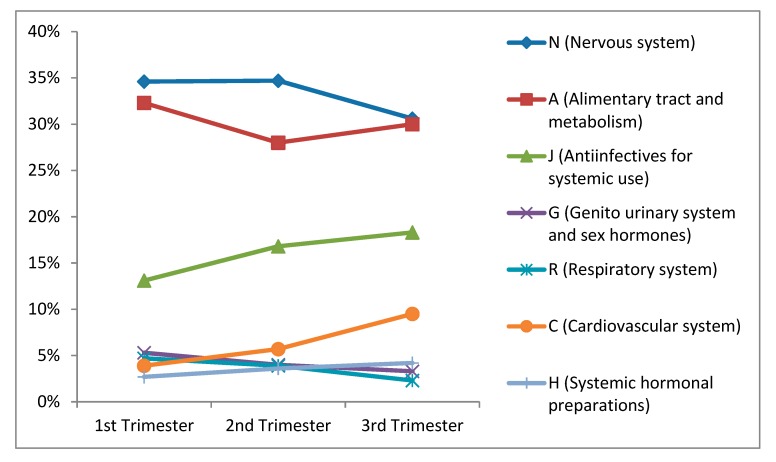
Medications most commonly used in each gestational trimester *, according Anatomical Therapeutic Classification System level 1. Pelotas (Brazil) Birth Cohort Study, 2015. * Excluding iron salts, folic acid, vitamins, and other minerals.

Figure 2 shows the proportion of medications used in each gestational trimester according to Anatomical Therapeutic Classification level 1. From the first to the third trimester of pregnancy, there was a slight decline in the proportion of use of drugs for the respiratory system and drugs for the genitourinary system and sex hormones. The proportion of drugs to the nervous system was very similar in the first and second trimesters, with a decline of just under 5% in the third trimester. The proportion of drugs for the alimentary tract and metabolism was very similar in all three trimesters, accounting for about 30% of the total drugs used in each trimester. There was a slight increase in the proportion of systemic hormone preparations over the three trimesters and a marked increase in the proportion of anti-infectives for systemic use (about 5% increase from the first to the third trimester) and cardiovascular system medications (5.5% increase from the first to the third trimester).

## 4. Discussion

The results of this work are part of a population-based birth cohort study of a medium-sized Brazilian municipality, with almost all births that occurred in the city in one year. The prevalence of drug use during pregnancy was 92.5%, a prevalence very similar to that found in other Brazilian and international studies [1,14,15] and slightly higher than that found in the study by Costa et al., conducted in Santo Antonio de Jesus (Bahia, Brazil), in 2017 [5]. There may be some heterogeneity in study results due to the different ways in which information is collected and analyzed (e.g., inclusion of minerals and vitamins [5,15], evaluation of the use of only over-the-counter or only prescription drugs [14], sampling at primary [5] or tertiary health care centers [15]), and differences in prescribing protocols for pregnant women across countries.

In this study, medication use was more frequent among white women with 12 years or more of formal education and among women who had a partner. Some Brazilian studies also showed more use of medicines during pregnancy among these groups [16,17]. International studies also show similar results for factors associated with medication use by pregnant women. In Argentina, a study showed higher drug use in older women with a high educational and socioeconomic status [18]. A study conducted in the USA and Canada showed that the use of prescription drugs increased with age and maternal education and was higher among non-Hispanic white women [7]. An Italian study also showed a greater chance of medication use among older, more educated women who reported health problems and who needed compulsory bed rest and/or hospitalization during pregnancy [19].

The number of prenatal consultations was also associated with medication use during pregnancy, as observed in the studies conducted by Andrade et al. [20] and Costa et al. [5]. However, in our study, only the variable number of acute and/or chronic diseases remained related to medication use after adjustment, a relationship also found in other studies [5,19,21].

Most of the medications used were prescription drugs, which reflects the almost universal Brazilian prenatal care. However, the prevalence of self-medication found in this study was 27.7%, higher than that found in the studies by Guerra et al. (12.2%) [22] and Costa et al. (13%) [5] and slightly lower than that found by Gomes et al. (33, 5%) [16].

We highlight the higher prevalence of self-medication in the first trimester, a critical period for congenital malformations, which may also be related to later pregnancy diagnosis. The factors that remained related to self-medication after adjustment were income, smoking, and parity. Women with higher incomes tend to have higher education and knowledge about possible risks to the fetus, as well as more access to medical care, which may result in lower self-medication among these pregnant women. The higher self-medication rate among multiparous women suggests more experience with common pregnancy symptoms and less concern about risks [23]. Among pregnant smokers, the greater presence of self-medication may be related to poorer health care and/or lower demand for medical care. Similar results regarding self-medication among multiparous women were found in the study by Guerra et al. in the city of Natal (Rio Grande do Norte, Brazil) [22].

Regarding the most commonly used drug classes, a Brazilian study also found a high prevalence of medication use for the gastrointestinal tract, which can be explained due to common pregnancy symptoms such as nausea, vomiting, and abdominal or pelvic pain [24].

Acetaminophen was the analgesic and antipyretic most commonly used by pregnant women in our study (about 22% by self-medication) and it is a widely used, non-steroidal, anti-inflammatory drug during pregnancy worldwide [25]. However, two recent systematic reviews have warned about its use during pregnancy [26,27]. Both studies found an association between paracetamol use during pregnancy and increased risk of attention deficit disorder, autism, and other neurodevelopmental disorders, although the authors point out that their findings should be taken with caution. In addition, there is also evidence showing that frequent use of acetaminophen, especially between the 20th and the 32nd weeks of gestation, is associated with an increased risk of asthma in children [28,29].

Another point that draws attention is the use of dipyrone (metamizole) without medical advice. Currently, this drug remains contraindicated during this period, although evidence has shown that exposure to dipyrone during pregnancy does not appear to increase the risk of congenital malformations or other adverse pregnancy outcomes [30,31,32]. Similarly, anti-inflammatory drugs, also largely consumed by self-medication in our sample, such as ibuprofen, are contraindicated mainly in the third trimester of pregnancy, as they can cause adverse effects such as delayed onset of labor, premature closure of the arterial duct, and pulmonary hypertension in the newborn [33,34]. Naphazoline, a vasoconstricting agent used as a nasal decongestant, can also cause premature closure of the arterial duct [35] and was widely used by self-medication by the pregnant women in our study.

The most commonly used chronic medications were levothyroxine, which is used for the treatment of hypothyroidism in pregnancy [36], and methyldopa, which still is one of the main drugs for the treatment of hypertension in pregnancy [37,38]. Although it is rare, methyldopa can cause hemolytic anemia and hepatotoxicity [39,40].

The use of acetylsalicylic acid in our study was low, which shows the tendency to disuse this drug as an analgesic. The use of acetylsalicylic acid should be avoided, especially at the end of pregnancy, with paracetamol being the most appropriate choice as an analgesic drug [41]. A meta-analysis has shown the beneficial effect of acetylsalicylic acid in preventing preeclampsia and fetal growth restriction, because it is administered until the sixteenth week of gestation [42]. The prevention of preeclampsia has been the main use of this drug lately [43].

One of the strengths of our work is the fact that is a population-based cohort study from a medium-sized city in Brazil, with a large sample size and good follow-up rates, thus covering information about a wide range of medications used by women in pregnancy, both for acute and chronic health problems. Studies that evaluated large drug databases in Brazil, such as the National Survey on Access, Use, and Promotion of Rational Use of Medicines in Brazil (PNAUM) [44] did not evaluate the use of medicines specifically in the pregnant women population. Another differential of this study was that the interviews were conducted not only at the time of delivery, but also during the prenatal period, thus qualifying data collection.

This study has some limitations. The time elapsed between medication use and the time of the interview can lead to a recall error and may underestimate the prevalence of medication use. However, this problem was minimized by supplementing the information with the information of the antenatal study. Self-reports can also generate errors, which could have been minimized by presenting prescriptions and the packaging of medications during the antenatal study, but which were unavailable at the time of the interviews at the maternity wards. Besides that, the evaluation of dosage and duration of medication use is limited, and some medications could not be classified due to lack of reason for use. However, we made a partial classification whenever it was possible, for example up to level 1 (anatomical main group) or 2 (therapeutic subgroup) of the ATC classification.

In Brazil, nurses usually prescribe ferrous sulfate and folic acid according to prenatal protocols [45], which were not evaluated in this study. However, if any other medication was prescribed by a nurse, it may have been referred to by the participants as not prescribed by a doctor or dentist and may have been classified as self-medication, thus representing another limitation of our study.

Even excluding the supplements recommended in the protocols of the Brazilian Ministry of Health for use during pregnancy, such as ferrous sulfate and folic acid [45], the almost universal practice of drug use as a mean of intervention in pregnancy is evident in this study [5]. It is important that all health care providers be aware of quaternary prevention in pregnancy, identifying women who are at risk of hypermedicalization. In the pregnancy context, use of health technologies supports reduction in maternal morbidity and mortality, but hypermedicalization can represent risks for women, fetuses, and newborns [46]. It is necessary to raise awareness of the risks of self-medication during pregnancy, preferably focusing on groups more prone to this practice. In addition, ensuring qualified prenatal care can help minimize unnecessary exposures and provide women with information about rational drug use during pregnancy.

## 5. Conclusions 

Medication use is a very common practice during pregnancy. This study describes the pattern of drug use among pregnant women in a population-based cohort study, showing a high prevalence of self-medication, including some drugs that are contraindicated during this period. Greater awareness of the risks of self-medication during pregnancy is required, focusing on groups more prone to this practice, as well as ensuring qualified multidisciplinary prenatal care.

## Figures and Tables

**Table 1:** Description of the study sample and prevalence of medication use and self-medication * according background variables. Pelotas (Brazil) Birth Cohort Study, 2015 (*N* = 4270).

	*N*	%	Medication Use			Self-Medication **		
			*N*	%	95% CI	*N*	%	95% CI
**Skin color**								
White	3005	70.5	2818	93.8	92.9–94.6	774	27.6	26.0–29.3
Black	680	15.9	604	88.8	86.4–91.2	158	26.2	22.7–29.7
Mixed/other	578	13.5	520	90	87.5–92.4	156	30.1	26.2–34.1
**Age**								
≤19	630	14.8	554	87.9	85.4–90.5	179	32.4	28.5–36.3
20–29	2021	47.3	1874	92.7	91.6–93.9	521	27.9	25.8–29.9
30–47	1618	37.9	1521	94	92.8–95.2	389	25.8	23.6–28.0
**Schooling (years)**								
0–4	394	9.2	330	83.8	80.1–87.4	103	31.2	26.2–36.2
5–8	1098	25.7	994	90.5	88.8–92.3	318	32.1	29.2–35.0
9–11	1463	34.3	1361	93	91.7–94.3	401	29.6	27.2–32.0
12 or more	1314	30.8	1263	96.1	95.1–97.2	267	21.3	19.0–23.6
Family monthly income (MW) ***								
≤1	548	12.8	471	85.9	83.0–88.9	141	30.1	25.9–34.2
11–3	2015	47.2	1851	91.9	90.7–93.1	566	30.7	28.6–32.8
3.1–6	1126	26.4	1069	94.9	93.7–96.2	287	27	24.3–29.6
6.1–10	316	7.4	306	96.8	94.9–98.8	53	17.4	13.1–21.7
>10	263	6.2	251	95.4	92.9–98.0	42	17.1	12.4–21.9
**Parity**								
1	2114	49.5	1965	93	91.9–94.0	493	25.2	23.3–27.2
2	1315	30.8	1229	93.5	92.1–94.8	330	27	24.5–29.5
3	472	11.1	433	91.7	89.2–94.2	147	34.1	29.6–38.6
4 or more	367	8.6	320	87.2	83.8–90.6	118	37	31.7–42.3
**Prenatal consultation**								
<6	603	14.5	532	88.2	85.6–90.8	177	33.3	29.3–37.4
6 or more	3552	85.5	3336	93.9	93.1–94.7	868	26.2	24.7–27.7
**Marital status**								
Living without a partner	619	14.5	550	88.9	86.4–91.3	189	34.5	30.5–38.5
Living with a partner	3650	85.5	3398	93.1	92.3–93.9	900	26.6	25.1–28.1
**Smoking**								
No	3553	83.2	3305	93	92.2–93.9	850	25.9	24.4–27.4
Yes	714	16.7	642	89.9	87.7–92.1	238	37.1	33.3–40.8
**Health problems in pregnancy (chronic or not)**								
No diseases	295	6.9	242	82	77.6–86.4	69	28.8	23.0–34.5
1	724	17	634	87.6	85.2–90.0	161	25.6	22.2–29.0
2	985	23.1	900	91.4	89.6–93.1	245	27.4	24.5–30.4
3	936	21.9	893	95.4	94.1–96.7	258	29.1	26.1–32.0
4 or more	1330	31.2	1280	96.2	95.2–97.3	356	27.9	25.4–30.3
**Hospitalization during current pregnancy**								
No	3429	80.3	3140	91.6	90.6–92.5	853	27.3	25.7–28.9
Yes	839	19.7	807	96.2	94.9–97.5	235	29.3	26.1–32.4
Pregnancy trimester at onset of prenatal care ****								
First	2067	54.8	1979	95.7	94.9–96.6	498	25.4	23.5–27.3
Second	1475	39.1	1349	91.5	90.0–92.9	405	30	27.6–32.5
Third	231	6.1	210	90	87.2–94.6	75	35.9	29.3–42.4
**Total**	4270	100	3949	92.5	91.7–93.3	1089	27.7	26.3–29.1

* Excluding iron salts, folic acid, vitamins, and other minerals; ** Subset of the group of women who used medications; *** MW: Minimum wages. Conversion rate R$ 788.00 = USD 200.00; **** Information based on prenatal charts. 3773 women.

**Table 2:** Prevalence ratios for medication use and self-medication * adjusted for background variables. Pelotas (Brazil) Birth Cohort Study, 2015.

Characteristics	Medication Use			Self-Medication **		
	Adjusted PR ***	95% CI	*p*-Value	Adjusted PR ***	95% CI	*p*-Value
**First level**						
**Skin color**			0.621			0.335
White	1			1		
Black	0.97	0.89–1.06		0.84	0.70–1.00	
Mixed/other	0.99	0.90–1.09		0.98	0.82–1.17	
**Age**			0.741			0.661
≤19	1			1		
20–29	1.03	0.93–1.14		0.97	0.81–1.16	
30–47	1.03	0.93–1.15		0.98	0.81–1.19	
**Schooling (years)**			0.07			0.580
0–4	1			1		
5–8	1.06	0.93–1.22		1.16	0.90–1.49	
9–11	1.09	0.95–1.25		1.17	0.91–1.51	
12 or more	1.13	0.98–1.30		0.98	0.73–1.31	
Family monthly income (MW) ****			0.624			0.044
≤1	1			1		
1.1–3	1.04	0.94–1.16		1.13	0.92–1.39	
3.1–6	1.06	0.94–1.19		1.07	0.84–1.36	
6.1–10	1.06	0.90–1.25		0.84	0.59–1.20	
>10	1.04	0.88–1.24		0.82	0.55–1.22	
**Marital Status**			0.594			0.184
Living without a partner	1			1		
Living with a partner	1.02	0.93–1.12		0.87	0.73–1.04	
**Second level**						
**Number of prenatal visits**			0.762			0.765
<6	1			1		
6 or more	1.02	0.91–1.13		0.96	0.79–1.17	
**Hospitalization during pregnancy**			0.586			0.818
No	1			1		
Yes	1.03	0.94–1.16		1.01	0.86–1.18	
**Onset of prenatal care**			0.357			0.052
First	1			1		
Second	0.96	0.89–1.03		1.12	0.97–1.28	
Third	0.97	0.83–1.13		1.24	0.95–1.61	
**Health problems**			0.021			0.479
0	1			1		
1	1.04	0.89–1.22		0.91	0.67–1.23	
2	1.09	0.94–1.27		0.95	0.71–1.27	
3	1.14	0.98–1.33		0.94	0.70–1.26	
4 or more	1.14	0.98–1.33		0.87	0.66–1.16	
**Smoking**			0.835			0.01
No	1			1		
Yes	1.01	0.92–1.12		1.27	1.08–1.51	
**Parity**			0.81			0.031
1	1			1		
2	1.01	0.94–1.09		1.05	0.91–1.22	
3	1.02	0.91–1.14		1.25	1.03–1.53	
4 or more	1.01	0.88–1.15		1.20	0.94–1.52	

* Excluding iron salts, folic acid, vitamins, and other minerals; ** Subset of the group of women who used medications; *** Only variables with *p* < 0.20 were maintained in the model, ensuring control for variables at the same and higher levels. **** Minimum wages conversion rate R$ 788.00 = USD 200.00.

**Table 3:** Medications most commonly used by pregnant women and self-medication proportion *, based on the Anatomical Therapeutic Classification System levels 1 and 5. (*N* = 14,064 medications). Pelotas (Brazil) Birth Cohort Study, 2015.

Therapeutic Groups	Medication Use		Self-Medication	
	*N*	%	*N*	%
A—Alimentary tract and metabolism	3964	30.9	542	13.7
Scopolamine	1037	26.2	56	5.4
Dimenhydrinate	922	23.3	126	13.7
Aluminum compounds	183	4.6	16	8.8
N—Nervous system	3676	28.6	871	23.7
Acetaminophen (paracetamol)	2654	72.2	559	21.1
Metamizole (dipyrone)	198	5.4	100	50.5
Scopolamin + acetaminophen	192	5.2	3	1.6
J—Anti-infectives for systemic use	2565	20	28	1.1
Antibiotic not specified	692	27.1	2	0.3
Cephalexin	605	23.7	0	0
Amoxicillin	405	15.9	12	3
C—Cardiovascular system	721	5.6	14	2
Methyldopa	309	42.9	2	0.7
Omega 3	187	25.9	8	4.3
Isoxsuprine	78	10.8	0	0
G—Genitourinary system and sex hormones	671	5.2	30	4.5
Progesterone	627	56	2	1.2
Vaginal cream not specified	122	11	1	0.8
Miconazole	111	9.9	1	0.9
R—Respiratory system	494	3.9	70	14.2
Meclizine	165	33.3	9	5.5
Albuterol	52	10.5	1	1.9
Naphazoline	45	9.1	24	53.3
H—Systemic hormonal preparations	305	2.4	1	0.3
Levothyroxine	243	79.7	1	0.4
Betamethasone	24	7.9	0	0
Prednisone	13	4.3	0	0
M—Musculo-skeletal system	240	1.9	84	35.4
Ibuprofen	104	43.7	39	37.9
Diclofenac	67	28.2	32	47.1
Ketoprofen	22	9.2	2	9.1
B—Blood and blood forming organs	94	0.7	11	11.5
Acetylsalicylic acid	82	85.4	9	11
Enoxaparin	3	3.1	0	0
Heparin	3	3.1	0	0
D—Dermatologicals	67	0.5	7	11.1
P—Antiparasitic products, insecticides	18	0.1	5	27.8
S—Sensory organs	17	0.1	3	16.7
L—Antineoplastic and immunomodulating agents	4	0.03	0	0
**Total**	12,836 **	100	1733	12.3 ***

* Excluding iron salts, folic acid, vitamins, and other minerals; ** Of the 14,064 drugs, 1228 could not be classified due to lack of reason for use (classification differs as to therapeutic use); *** A total of 27 drugs unclassified due to lack of reason for use.
